# Supplementary material for: Base Editors for Citrus Gene Editing
Source: Front Genome Ed. 2022 Feb 28;4:852867. doi: 10.3389/fgeed.2022.852867 (PMC8919994; doi:10.3389/fgeed.2022.852867)
Supplement: Supplementary file 2 [file Table1.DOCX]

**PC-CmYLCV-ABE8e-LOB1TATA vector**

**The vector contains a GFP expression cassette which is not shown here.**

**Green: CsU6 promoter; grey: tRNA; red: gRNA for TATA box editing; Blue: optimized gRNA scaffold; purple: CmYLCV promoter; Yellow highlight: ABE8e; orange: HSP18.2 terminator.**

**GCGCTCAGGAGCCGGTTGAATTTGATTGTTGTTTGATGTTTAGGTATGCTTACAATTTTTTACTAAATAAGGTAATAATTGTTCCTTTTATTTTATTCATTTAGCTTGGTACTTGTCGTAATCTATCTCCGTCAATGTCAGCTTCTTCTGTGTCGGAAGAAGCTGAAAAATGTTGTTGAAGGCCCTTTCATTTATTATTATCATTATTATGTTTAATTTTGTTCGGCAAATGTGTACTAATTTGGTTCGAATCGTTCGATCTCATAATACAAACATGTAATGAGTTATTTATGACGTCATGGATGCGGTTAGTTTGCGTGGTCTGAAATTTCAACCAAATTATTCAATCATGGTGGTGGCCGGCTGGTGCCTATCCCTTGATTGAAATATGCAATTAAAATTCTCGTAATAATGTTGATTTGTTCTATATACTTGAATTGATGGTATTAATTATTGTTATAAAAGCACTAACTTGTTTGAGAAAGGGGATAACTAAAAGGTAATAATAATAATAATAAAATAAAGAAAAGGCCCAACACATGGGCGCCCTATCTGTTGGGCCAAATGCCATAAGAGGATCCAGCTAGCCCGTTGAAGAAAATCCCACATCGAAAGAAAAAACTGAATAAACATGGTCTATATATACAAGGACTCCCAGGTTGGTTGAACAAAGCACCAGTGGTCTAGTGGTAGAATAGTACCCTGCCACGGTACAGACCCGGGTTCGATTCCCGGCTGGTGCAGTTTATATAGAGAAAGGAAAGTTTAAGAGCTATGCTGGAAACAGCATAGCAAGTTTAAATAAGGCTAGTCCGTTATCAACTTGAAAAAGTGGCACCGAGTCGGTGCAACAAAGCACCAGTGGTCTAGTGGTAGAATAGTACCCTGCCACGGTACAGACCCGGGTTCGATTCCCGGCTGGTGCATTTTTTTTTCCCCTGTTTTTTCCTAATTAGATTTCTTTCGGAGCTGTTCGAAGAACATGTTTTTGCCGTCTGACTCGTTTGTTATTGCCGTTCTAGTTAGTTTCAGCTATTGGTTTTCGGTTTCTTTCTGTTTTGAAGGGTAATAGTTTGGAAAATATAATACAAATTAATTTAGGATTAAAAAGTAATAAAACAATATTATCCCTGCAGGCGATCGGCGCGCCAGATTTGCCTTTTCAATTTCAGAAAGAATGCTAACCCACAGATGGTTAGAGAGGCTTACGCAGCAGGTATCATCAAGACGATCTACCCGAGCAATAATCTCCAGGAAATCAAATACCTTCCCAAGAAGGTTAAAGATGCAGTCAAAAGATTCAGGACTAACTGCATCAAGAACACAGAGAAAGATATATTTCTCAAGATCAGAAGTACTATTCCAGTATGGACGATTCAAGGCTTGCTTCACAAACCAAGGCAAGTAATAGAGATTGGAGTCTCTAAAAAGGTAGTTCCCACTGAATCAAAGGCCATGGAGTCAAAGATTCAAATAGAGGACCTAACAGAACTCGCCGTAAAGACTGGCGAACAGTTCATACAGAGTCTCTTACGACTCAATGACAAGAAGAAAATCTTCGTCAACATGGTGGAGCACGACACACTTGTCTACTCCAAAAATATCAAAGATACAGTCTCAGAAGACCAAAGGGCAATTGAGACTTTTCAACAAAGGGTAATATCCGGATGGCAGACATACTGTCCCACAAATGAAGATGGAATCTGTAAAAGAAAACGCGTGAAATAATGCGTCTGACAAAGGTTAGGTCGGCTGCCTTTAATCAATACCAAAGTGGTCCCTACCACGATGGAAAAACTGTGCAGTCGGTTTGGCTTTTTCTGACGAACAAATAAGATTCGTGGCCGACAGGTGGGGGTCCACCATGTGAAGGCATCTTCAGACTCCAATAATGGAGCAATGACGTAAGGGCTTACGAAATAAGTAAGGGTAGTTTGGGAAATGTCCACTCACCCGTCAGTCTATAAATACTTAGCCCCTCCCTCATTGTTAAGGGAGCAAAATCTCAGAGAGATAGTCCTAGAGAGAGAAAGAGAGCAAGTAGCCTAGAAGTAGTCAAGGCGGCGAAGTATTCAGGCACGTGGCCAGGAAGAAGAAAAGCCAAGACGACGAAAACAGGTAAGAGCTAAGCTTTCTAGAatgaaacggacagccgacggaagcgagttcgagtcaccaaagaagaagcggaaagtctctgaggtggagttttcccacgagtactggatgagacatgccctgaccctggccaagagggcacgggatgagagggaggtgcctgtgggagccgtgctggtgctgaacaatagagtgatcggcgagggctggaacagagccatcggcctgcacgacccaacagcccatgccgaaattatggccctgagacagggcggcctggtcatgcagaactacagactgattgacgccaccctgtacgtgacattcgagccttgcgtgatgtgcgccggcgccatgatccactctaggatcggccgcgtggtgtttggcgtgaggaactcaaaaagaggcgccgcaggctccctgatgaacgtgctgaactaccccggcatgaatcaccgcgtcgaaattaccgagggaatcctggcagatgaatgtgccgccctgctgtgcgatttctatcggatgcctagacaggtgttcaatgctcagaagaaggcccagagctccatcaactccggaggatctagcggaggctcctctggctctgagacacctggcacaagcgagagcgcaacacctgaaagcagcgggggcagcagcggggggtcagacaagaagtacagcatcGgcctggcTATCGGAACTAACTCTGTGGGATGGGCTGTGATCACCGATGAGTACAAGGTGCCATCTAAGAAGTTCAAGGTTCTCGGAAACACCGATAGGCACTCTATCAAGAAAAACCTTATCGGTGCTCTCCTCTTCGATTCTGGTGAAACTGCTGAGGCTACCAGACTCAAGAGAACCGCTAGAAGAAGGTACACCAGAAGAAAGAACAGGATCTGCTACCTCCAAGAGATCTTCTCTAACGAGATGGCTAAAGTGGATGATTCATTCTTCCACAGGCTCGAAGAGTCATTCCTCGTGGAAGAAGATAAGAAGCACGAGAGGCACCCTATCTTCGGAAACATCGTTGATGAGGTGGCATACCACGAGAAGTACCCTACTATCTACCACCTCAGAAAGAAGCTCGTTGATTCTACTGATAAGGCTGATCTCAGGCTCATCTACCTCGCTCTCGCTCACATGATCAAGTTCAGAGGACACTTCCTCATCGAGGGTGATCTCAACCCTGATAACTCTGATGTGGATAAGTTGTTCATCCAGCTCGTGCAGACCTACAACCAGCTTTTCGAAGAGAACCCTATCAACGCTTCAGGTGTGGATGCTAAGGCTATCCTCTCTGCTAGGCTCTCTAAGTCAAGAAGGCTTGAGAACCTCATTGCTCAGCTCCCTGGTGAGAAGAAGAACGGACTTTTCGGAAACTTGATCGCTCTCTCTCTCGGACTCACCCCTAACTTCAAGTCTAACTTCGATCTCGCTGAGGATGCAAAGCTCCAGCTCTCAAAGGATACCTACGATGATGATCTCGATAACCTCCTCGCTCAGATCGGAGATCAGTACGCTGATTTGTTCCTCGCTGCTAAGAACCTCTCTGATGCTATCCTCCTCAGTGATATCCTCAGAGTGAACACCGAGATCACCAAGGCTCCACTCTCAGCTTCTATGATCAAGAGATACGATGAGCACCACCAGGATCTCACACTTCTCAAGGCTCTTGTTAGACAGCAGCTCCCAGAGAAGTACAAAGAGATTTTCTTCGATCAGTCTAAGAACGGATACGCTGGTTACATCGATGGTGGTGCATCTCAAGAAGAGTTCTACAAGTTCATCAAGCCTATCCTCGAGAAGATGGATGGAACCGAGGAACTCCTCGTGAAGCTCAATAGAGAGGATCTTCTCAGAAAGCAGAGGACCTTCGATAACGGATCTATCCCTCATCAGATCCACCTCGGAGAGTTGCACGCTATCCTTAGAAGGCAAGAGGATTTCTACCCATTCCTCAAGGATAACAGGGAAAAGATTGAGAAGATTCTCACCTTCAGAATCCCTTACTACGTGGGACCTCTCGCTAGAGGAAACTCAAGATTCGCTTGGATGACCAGAAAGTCTGAGGAAACCATCACCCCTTGGAACTTCGAAGAGGTGGTGGATAAGGGTGCTAGTGCTCAGTCTTTCATCGAGAGGATGACCAACTTCGATAAGAACCTTCCAAACGAGAAGGTGCTCCCTAAGCACTCTTTGCTCTACGAGTACTTCACCGTGTACAACGAGTTGACCAAGGTTAAGTACGTGACCGAGGGAATGAGGAAGCCTGCTTTTTTGTCAGGTGAGCAAAAGAAGGCTATCGTTGATCTCTTGTTCAAGACCAACAGAAAGGTGACCGTGAAGCAGCTCAAAGAGGATTACTTCAAGAAAATCGAGTGCTTCGATTCAGTTGAGATTTCTGGTGTTGAGGATAGGTTCAACGCATCTCTCGGAACCTACCACGATCTCCTCAAGATCATTAAGGATAAGGATTTCTTGGATAACGAGGAAAACGAGGATATCTTGGAGGATATCGTTCTTACCCTCACCCTCTTTGAAGATAGAGAGATGATTGAAGAAAGGCTCAAGACCTACGCTCATCTCTTCGATGATAAGGTGATGAAGCAGTTGAAGAGAAGAAGATACACTGGTTGGGGAAGGCTCTCAAGAAAGCTCATTAACGGAATCAGGGATAAGCAGTCTGGAAAGACAATCCTTGATTTCCTCAAGTCTGATGGATTCGCTAACAGAAACTTCATGCAGCTCATCCACGATGATTCTCTCACCTTTAAAGAGGATATCCAGAAGGCTCAGGTTTCAGGACAGGGTGATAGTCTCCATGAGCATATCGCTAACCTCGCTGGATCTCCTGCAATCAAGAAGGGAATCCTCCAGACTGTGAAGGTTGTGGATGAGTTGGTGAAGGTGATGGGAAGGCATAAGCCTGAGAACATCGTGATCGAAATGGCTAGAGAGAACCAGACCACTCAGAAGGGACAGAAGAACTCTAGGGAAAGGATGAAGAGGATCGAGGAAGGTATCAAAGAGCTTGGATCTCAGATCCTCAAAGAGCACCCTGTTGAGAACACTCAGCTCCAGAATGAGAAGCTCTACCTCTACTACCTCCAGAACGGAAGGGATATGTATGTGGATCAAGAGTTGGATATCAACAGGCTCTCTGATTACGATGTTGATCATATCGTGCCACAGTCATTCTTGAAGGATGATTCTATCGATAACAAGGTGCTCACCAGGTCTGATAAGAACAGGGGTAAGAGTGATAACGTGCCAAGTGAAGAGGTTGTGAAGAAAATGAAGAACTATTGGAGGCAGCTCCTCAACGCTAAGCTCATCACTCAGAGAAAGTTCGATAACTTGACTAAGGCTGAGAGGGGAGGACTCTCTGAATTGGATAAGGCAGGATTCATCAAGAGGCAGCTTGTGGAAACCAGGCAGATCACTAAGCACGTTGCACAGATCCTCGATTCTAGGATGAACACCAAGTACGATGAGAACGATAAGTTGATCAGGGAAGTGAAGGTTATCACCCTCAAGTCAAAGCTCGTGTCTGATTTCAGAAAGGATTTCCAATTCTACAAGGTGAGGGAAATCAACAACTACCACCACGCTCACGATGCTTACCTTAACGCTGTTGTTGGAACCGCTCTCATCAAGAAGTATCCTAAGCTCGAGTCAGAGTTCGTGTACGGTGATTACAAGGTGTACGATGTGAGGAAGATGATCGCTAAGTCTGAGCAAGAGATCGGAAAGGCTACCGCTAAGTATTTCTTCTACTCTAACATCATGAATTTCTTCAAGACCGAGATTACCCTCGCTAACGGTGAGATCAGAAAGAGGCCACTCATCGAGACAAACGGTGAAACAGGTGAGATCGTGTGGGATAAGGGAAGGGATTTCGCTACCGTTAGAAAGGTGCTCTCTATGCCACAGGTGAACATCGTTAAGAAAACCGAGGTGCAGACCGGTGGATTCTCTAAAGAGTCTATCCTCCCTAAGAGGAACTCTGATAAGCTCATTGCTAGGAAGAAGGATTGGGACCCTAAGAAATACGGTGGTTTCGATTCTCCTACCGTGGCTTACTCTGTTCTCGTTGTGGCTAAGGTTGAGAAGGGAAAGAGTAAGAAGCTCAAGTCTGTTAAGGAACTTCTCGGAATCACTATCATGGAAAGGTCATCTTTCGAGAAGAACCCAATCGATTTCCTCGAGGCTAAGGGATACAAAGAGGTTAAGAAGGATCTCATCATCAAGCTCCCAAAGTACTCACTCTTCGAACTCGAGAACGGTAGAAAGAGGATGCTCGCTTCTGCTGGTGAGCTTCAAAAGGGAAACGAGCTTGCTCTCCCATCTAAGTACGTTAACTTTCTTTACCTCGCTTCTCACTACGAGAAGTTGAAGGGATCTCCAGAAGATAACGAGCAGAAGCAACTTTTCGTTGAGCAGCACAAGCACTACTTGGATGAGATCATCGAGCAGATCTCTGAGTTCTCTAAAAGGGTGATCCTCGCTGATGCAAACCTCGATAAGGTGTTGTCTGCTTACAACAAGCACAGAGATAAGCCTATCAGGGAACAGGCAGAGAACATCATCCATCTCTTCACCCTTACCAACCTCGGTGCTCCTGCTGCTTTCAAGTACTTCGATACAACCATCGATAGGAAGAGATACACCTCTACCAAAGAAGTGCTCGATGCTACCCTCATCCATCAGTCTATCACTGGACTCTACGAGACTAGGATCGATCTCTCACAGCTCGGTGGTGATTCAAGGGCTGATgaattcgagcccaagaagaagagGAAAGTCTAAATATGAAGATGAAGATGAAATATTTGGTGTGTCAAATAAAAAGCTTGTGTGCTTAAGTTTGTGTTTTTTTCTTGGCTTGTTGTGTTATGAATTTGTGGCTTTTTCTAATATTAAATGAATGTAAGATCACATTATAATGAATAAACAAATGTTTCTATAATCCATTGTGAATGTTTTGTTGGATCTCTTCTGCAGCATATAACTACTGTATGTGCTATGGTATGGACTATGGAATATGATTAAAGATAAG**

PC-CmYLCV-CBE-2XCsALS

CsU6 promoter; gRNA scaffold; CsALS gRNA; CmYLCV promoter; A3A; Linker; RAD51-DBD; nCas9; nucleoplasmin NLS; UGI; SV40 NLS

GCGCTCAGGAGCCGGTTGAATTTGATTGTTGTTTGATGTTTAGGTATGCTTACAATTTTTTACTAAATAAGGTAATAATTGTTCCTTTTATTTTATTCATTTAGCTTGGTACTTGTCGTAATCTATCTCCGTCAATGTCAGCTTCTTCTGTGTCGGAAGAAGCTGAAAAATGTTGTTGAAGGCCCTTTCATTTATTATTATCATTATTATGTTTAATTTTGTTCGGCAAATGTGTACTAATTTGGTTCGAATCGTTCGATCTCATAATACAAACATGTAATGAGTTATTTATGACGTCATGGATGCGGTTAGTTTGCGTGGTCTGAAATTTCAACCAAATTATTCAATCATGGTGGTGGCCGGCTGGTGCCTATCCCTTGATTGAAATATGCAATTAAAATTCTCGTAATAATGTTGATTTGTTCTATATACTTGAATTGATGGTATTAATTATTGTTATAAAAGCACTAACTTGTTTGAGAAAGGGGATAACTAAAAGGTAATAATAATAATAATAAAATAAAGAAAAGGCCCAACACATGGGCGCCCTATCTGTTGGGCCAAATGCCATAAGAGGATCCAGCTAGCCCGTTGAAGAAAATCCCACATCGAAAGAAAAAACTGAATAAACATGGTCTATATATACAAGGACTCCCAGGTTGGTTGAACAAAGCACCAGTGGTCTAGTGGTAGAATAGTACCCTGCCACGGTACAGACCCGGGTTCGATTCCCGGCTGGTGCA(tRNA)CAGGTCCC**T**CGGAGGATGATGTTTAAGAGCTATGCTGGAAACAGCATAGCAAGTTTAAATAAGGCTAGTCCGTTATCAACTTGAAAAAGTGGCACCGAGTCGGTGCAACAAAGCACCAGTGGTCTAGTGGTAGAATAGTACCCTGCCACGGTACAGACCCGGGTTCGATTCCCGGCTGGTGCA(tRNA)CAGGTCCC**G**CGGAGGATGATGTTTAAGAGCTATGCTGGAAACAGCATAGCAAGTTTAAATAAGGCTAGTCCGTTATCAACTTGAAAAAGTGGCACCGAGTCGGTGCAACAAAGCACCAGTGGTCTAGTGGTAGAATAGTACCCTGCCACGGTACAGACCCGGGTTCGATTCCCGGCTGGTGCA(tRNA)**TTTTTTTTT**CCCCTGTTTTTTCCTAATTAGATTTCTTTCGGAGCTGTTCGAAGAACATGTTTTTGCCGTCTGACTCGTTTGTTATTGCCGTTCTAGTTAGTTTCAGCTATTGGTTTTCGGTTTCTTTCTGTTTTGAAGGGTAATAGTTTGGAAAATATAATACAAATTAATTTAGGATTAAAAAGTAATAAAACAATATTATCCCTGCAGGCGATCGGCGCGCCAGATTTGCCTTTTCAATTTCAGAAAGAATGCTAACCCACAGATGGTTAGAGAGGCTTACGCAGCAGGTATCATCAAGACGATCTACCCGAGCAATAATCTCCAGGAAATCAAATACCTTCCCAAGAAGGTTAAAGATGCAGTCAAAAGATTCAGGACTAACTGCATCAAGAACACAGAGAAAGATATATTTCTCAAGATCAGAAGTACTATTCCAGTATGGACGATTCAAGGCTTGCTTCACAAACCAAGGCAAGTAATAGAGATTGGAGTCTCTAAAAAGGTAGTTCCCACTGAATCAAAGGCCATGGAGTCAAAGATTCAAATAGAGGACCTAACAGAACTCGCCGTAAAGACTGGCGAACAGTTCATACAGAGTCTCTTACGACTCAATGACAAGAAGAAAATCTTCGTCAACATGGTGGAGCACGACACACTTGTCTACTCCAAAAATATCAAAGATACAGTCTCAGAAGACCAAAGGGCAATTGAGACTTTTCAACAAAGGGTAATATCCGGATGGCAGACATACTGTCCCACAAATGAAGATGGAATCTGTAAAAGAAAACGCGTGAAATAATGCGTCTGACAAAGGTTAGGTCGGCTGCCTTTAATCAATACCAAAGTGGTCCCTACCACGATGGAAAAACTGTGCAGTCGGTTTGGCTTTTTCTGACGAACAAATAAGATTCGTGGCCGACAGGTGGGGGTCCACCATGTGAAGGCATCTTCAGACTCCAATAATGGAGCAATGACGTAAGGGCTTACGAAATAAGTAAGGGTAGTTTGGGAAATGTCCACTCACCCGTCAGTCTATAAATACTTAGCCCCTCCCTCATTGTTAAGGGAGCAAAATCTCAGAGAGATAGTCCTAGAGAGAGAAAGAGAGCAAGTAGCCTAGAAGTAGTCAAGGCGGCGAAGTATTCAGGCACGTGGCCAGGAAGAAGAAAAGCCAAGACGACGAAAACAGGTAAGAGCTAAGCTTtctagaATGAAAAGAACAGCTGACGGCAGTgagttcGAGAGCCCCAAGAAGAAGCGTAAAGTTGAGGCAAGCCCAGCTTCAGGCCCCAGACACCTCATGGACCCACACATCTTCACATCAAATTTCAATAATGGAATTGGGAGACATAAAACATATCTATGCTACGAAGTGGAGCGTCTCGATAATGGCACCAGCGTCAAGATGGACCAACATCGTGGATTTCTGCATAATCAAGCCAAAAACCTGCTATGTGGGTTTTACGGACGACATGCAGAGTTACGTTTTCTTGACCTCGTGCCTTCACTACAGCTTGATCCCGCCCAAATATATCGAGTTACCTGGTTTATTTCCTGGTCTCCATGTTTCTCTTGGGGGTGTGCTGGGGAAGTCCGAGCCTTTTTACAGGAGAATACACATGTCAGGTTGAGGATCTTTGCTGCCAGAATCTATGATTATGATCCCCTTTACAAGGAAGCACTCCAGATGTTAAGAGACGCTGGCGCACAAGTGAGCATTATGACTTATGACGAGTTCAAACATTGCTGGGATACCTTTGTCGATCATCAAGGATGTCCTTTTCAGCCTTGGGACGGTCTGGACGAACATTCCCAGGCATTAAGCGGACGTTTGCGTGCTATACTTCAAAATCAAGGTAACTCCGGCGGGTCATCAGGTGGCAGCAGTGGATCTGAGACCCCCGGTACTTCTGAATCAGCAACTCCTGAATCCTCTGGTGGCTCTTCTGGGGGCAGCGCAATGCAAATGCAGCTTGAAGCAAACGCAGATACTTCCGTTGAAGAAGAGTCTTTCGGACCCCAGCCAATATCCCGACTTGAGCAGTGCGGCATAAATGCTAATGACGTAAAAAAACTGGAGGAGGCTGGGTTCCATACTGTCGAAGCAGTGGCCTACGCACCTAAGAAAGAATTAATAAATATCAAGGGCATATCAGAGGCAAAGGCTGACAAAATACTGGCTGAGGCCGCCAAGTTAGTCCCTATGGGATTTACCACTGCCACCGAGTTCCATCAGCGTCGATCAGAGATTATACAAATCACAACTGGATCCAAAGAACTAGATAAACTGTTACAAgtcgactccggaggatctaggtcgactccggaggatctagcggaggctcctctggctctgagacacctggcacaagcgagagcgcaacacctgaaagcagcgggggcagcagcggggggtcagacaagaagtacagcatcGgcctggcTATCGGAACTAACTCTGTGGGATGGGCTGTGATCACCGATGAGTACAAGGTGCCATCTAAGAAGTTCAAGGTTCTCGGAAACACCGATAGGCACTCTATCAAGAAAAACCTTATCGGTGCTCTCCTCTTCGATTCTGGTGAAACTGCTGAGGCTACCAGACTCAAGAGAACCGCTAGAAGAAGGTACACCAGAAGAAAGAACAGGATCTGCTACCTCCAAGAGATCTTCTCTAACGAGATGGCTAAAGTGGATGATTCATTCTTCCACAGGCTCGAAGAGTCATTCCTCGTGGAAGAAGATAAGAAGCACGAGAGGCACCCTATCTTCGGAAACATCGTTGATGAGGTGGCATACCACGAGAAGTACCCTACTATCTACCACCTCAGAAAGAAGCTCGTTGATTCTACTGATAAGGCTGATCTCAGGCTCATCTACCTCGCTCTCGCTCACATGATCAAGTTCAGAGGACACTTCCTCATCGAGGGTGATCTCAACCCTGATAACTCTGATGTGGATAAGTTGTTCATCCAGCTCGTGCAGACCTACAACCAGCTTTTCGAAGAGAACCCTATCAACGCTTCAGGTGTGGATGCTAAGGCTATCCTCTCTGCTAGGCTCTCTAAGTCAAGAAGGCTTGAGAACCTCATTGCTCAGCTCCCTGGTGAGAAGAAGAACGGACTTTTCGGAAACTTGATCGCTCTCTCTCTCGGACTCACCCCTAACTTCAAGTCTAACTTCGATCTCGCTGAGGATGCAAAGCTCCAGCTCTCAAAGGATACCTACGATGATGATCTCGATAACCTCCTCGCTCAGATCGGAGATCAGTACGCTGATTTGTTCCTCGCTGCTAAGAACCTCTCTGATGCTATCCTCCTCAGTGATATCCTCAGAGTGAACACCGAGATCACCAAGGCTCCACTCTCAGCTTCTATGATCAAGAGATACGATGAGCACCACCAGGATCTCACACTTCTCAAGGCTCTTGTTAGACAGCAGCTCCCAGAGAAGTACAAAGAGATTTTCTTCGATCAGTCTAAGAACGGATACGCTGGTTACATCGATGGTGGTGCATCTCAAGAAGAGTTCTACAAGTTCATCAAGCCTATCCTCGAGAAGATGGATGGAACCGAGGAACTCCTCGTGAAGCTCAATAGAGAGGATCTTCTCAGAAAGCAGAGGACCTTCGATAACGGATCTATCCCTCATCAGATCCACCTCGGAGAGTTGCACGCTATCCTTAGAAGGCAAGAGGATTTCTACCCATTCCTCAAGGATAACAGGGAAAAGATTGAGAAGATTCTCACCTTCAGAATCCCTTACTACGTGGGACCTCTCGCTAGAGGAAACTCAAGATTCGCTTGGATGACCAGAAAGTCTGAGGAAACCATCACCCCTTGGAACTTCGAAGAGGTGGTGGATAAGGGTGCTAGTGCTCAGTCTTTCATCGAGAGGATGACCAACTTCGATAAGAACCTTCCAAACGAGAAGGTGCTCCCTAAGCACTCTTTGCTCTACGAGTACTTCACCGTGTACAACGAGTTGACCAAGGTTAAGTACGTGACCGAGGGAATGAGGAAGCCTGCTTTTTTGTCAGGTGAGCAAAAGAAGGCTATCGTTGATCTCTTGTTCAAGACCAACAGAAAGGTGACCGTGAAGCAGCTCAAAGAGGATTACTTCAAGAAAATCGAGTGCTTCGATTCAGTTGAGATTTCTGGTGTTGAGGATAGGTTCAACGCATCTCTCGGAACCTACCACGATCTCCTCAAGATCATTAAGGATAAGGATTTCTTGGATAACGAGGAAAACGAGGATATCTTGGAGGATATCGTTCTTACCCTCACCCTCTTTGAAGATAGAGAGATGATTGAAGAAAGGCTCAAGACCTACGCTCATCTCTTCGATGATAAGGTGATGAAGCAGTTGAAGAGAAGAAGATACACTGGTTGGGGAAGGCTCTCAAGAAAGCTCATTAACGGAATCAGGGATAAGCAGTCTGGAAAGACAATCCTTGATTTCCTCAAGTCTGATGGATTCGCTAACAGAAACTTCATGCAGCTCATCCACGATGATTCTCTCACCTTTAAAGAGGATATCCAGAAGGCTCAGGTTTCAGGACAGGGTGATAGTCTCCATGAGCATATCGCTAACCTCGCTGGATCTCCTGCAATCAAGAAGGGAATCCTCCAGACTGTGAAGGTTGTGGATGAGTTGGTGAAGGTGATGGGAAGGCATAAGCCTGAGAACATCGTGATCGAAATGGCTAGAGAGAACCAGACCACTCAGAAGGGACAGAAGAACTCTAGGGAAAGGATGAAGAGGATCGAGGAAGGTATCAAAGAGCTTGGATCTCAGATCCTCAAAGAGCACCCTGTTGAGAACACTCAGCTCCAGAATGAGAAGCTCTACCTCTACTACCTCCAGAACGGAAGGGATATGTATGTGGATCAAGAGTTGGATATCAACAGGCTCTCTGATTACGATGTTGATCATATCGTGCCACAGTCATTCTTGAAGGATGATTCTATCGATAACAAGGTGCTCACCAGGTCTGATAAGAACAGGGGTAAGAGTGATAACGTGCCAAGTGAAGAGGTTGTGAAGAAAATGAAGAACTATTGGAGGCAGCTCCTCAACGCTAAGCTCATCACTCAGAGAAAGTTCGATAACTTGACTAAGGCTGAGAGGGGAGGACTCTCTGAATTGGATAAGGCAGGATTCATCAAGAGGCAGCTTGTGGAAACCAGGCAGATCACTAAGCACGTTGCACAGATCCTCGATTCTAGGATGAACACCAAGTACGATGAGAACGATAAGTTGATCAGGGAAGTGAAGGTTATCACCCTCAAGTCAAAGCTCGTGTCTGATTTCAGAAAGGATTTCCAATTCTACAAGGTGAGGGAAATCAACAACTACCACCACGCTCACGATGCTTACCTTAACGCTGTTGTTGGAACCGCTCTCATCAAGAAGTATCCTAAGCTCGAGTCAGAGTTCGTGTACGGTGATTACAAGGTGTACGATGTGAGGAAGATGATCGCTAAGTCTGAGCAAGAGATCGGAAAGGCTACCGCTAAGTATTTCTTCTACTCTAACATCATGAATTTCTTCAAGACCGAGATTACCCTCGCTAACGGTGAGATCAGAAAGAGGCCACTCATCGAGACAAACGGTGAAACAGGTGAGATCGTGTGGGATAAGGGAAGGGATTTCGCTACCGTTAGAAAGGTGCTCTCTATGCCACAGGTGAACATCGTTAAGAAAACCGAGGTGCAGACCGGTGGATTCTCTAAAGAGTCTATCCTCCCTAAGAGGAACTCTGATAAGCTCATTGCTAGGAAGAAGGATTGGGACCCTAAGAAATACGGTGGTTTCGATTCTCCTACCGTGGCTTACTCTGTTCTCGTTGTGGCTAAGGTTGAGAAGGGAAAGAGTAAGAAGCTCAAGTCTGTTAAGGAACTTCTCGGAATCACTATCATGGAAAGGTCATCTTTCGAGAAGAACCCAATCGATTTCCTCGAGGCTAAGGGATACAAAGAGGTTAAGAAGGATCTCATCATCAAGCTCCCAAAGTACTCACTCTTCGAACTCGAGAACGGTAGAAAGAGGATGCTCGCTTCTGCTGGTGAGCTTCAAAAGGGAAACGAGCTTGCTCTCCCATCTAAGTACGTTAACTTTCTTTACCTCGCTTCTCACTACGAGAAGTTGAAGGGATCTCCAGAAGATAACGAGCAGAAGCAACTTTTCGTTGAGCAGCACAAGCACTACTTGGATGAGATCATCGAGCAGATCTCTGAGTTCTCTAAAAGGGTGATCCTCGCTGATGCAAACCTCGATAAGGTGTTGTCTGCTTACAACAAGCACAGAGATAAGCCTATCAGGGAACAGGCAGAGAACATCATCCATCTCTTCACCCTTACCAACCTCGGTGCTCCTGCTGCTTTCAAGTACTTCGATACAACCATCGATAGGAAGAGATACACCTCTACCAAAGAAGTGCTCGATGCTACCCTCATCCATCAGTCTATCACTGGACTCTACGAGACTAGGATCGATCTCTCACAGCTCGGTGGTGATTCAAGGGCTGATgaattcAAGAGACCCGCAGCAACCAAGAAggcagggcaagcaaagaagaagaagacgcgtgactccggcggcagcaccaacctgtccgacatcatcgagaaggagacgggcaagcaactcgtgatccaggagagcatcctcatgctgccagaggaggtggaggaggtcatcggcaacaagccagagtccgacatcctggtgcacaccgcctacgacgagtccaccgacgagaacgtcatgctcctgaccagcgacgccccagagtacaagccatgggccctcgtcatccaggacagcaacggggagaacaagatcAAGATGCTGTCGGGGGGGAGCCaattcgagCCCAAGAAGAAGAGGAAAGTCTAAATATGAAGATGAAGATGAAATATTTGGTGTGTCAAATAAAAAGCTTGTGTGCTTAAGTTTGTGTTTTTTTCTTGGCTTGTTGTGTTATGAATTTGTGGCTTTTTCTAATATTAAATGAATGTAAGATCACATTATAATGAATAAACAAATGTTTCTATAATCCATTGTGAATGTTTTGTTGGATCTCTTCTGCAGCATATAACTACTGTATGTGCTATGGTATGGACTATGGAATATGATTAAAGATAAG(HSP18.2 terminator)
