# Supplementary material for: Base Editors for Citrus Gene Editing
Source: Front Genome Ed. 2022 Feb 28;4:852867. doi: 10.3389/fgeed.2022.852867 (PMC8919994; doi:10.3389/fgeed.2022.852867)
Supplement: Supplementary file 3 [file Table3.DOCX]

|  | **Sequence** | **Locus** | **Gene** | **Region** | **Off-target edits in Hamlin mutant** | **Off-target edits in Grapefruit**  **mutant** |
| --- | --- | --- | --- | --- | --- | --- |
| **#** | **For LOB1:** |  |  |  |  |  |
| 1 | **A**TTTATATA**A**AGAAA**AA**AAA**TGG** | chrUn:+30450255 |  | Intergenic | No | No |
| 2 | GTTTATA**A**A**A**AGAAA**AA**AAA**AGG** | chr1:-8435697 |  | Intergenic | No | No |
| 3 | GTTTA**A**A**A**A**A**A**A**AAAGGAAA**AGG** | chr1:-26798360 |  | Intergenic | No | No |
| 4 | **T**T**A**TATATA**T**AGAAAG**A**AAA**AGG** | chr2:+4106970 |  | Intergenic | No | No |
| 5 | **T**T**A**TAT**G**TAGA**A**AAAGGAAA**AGG** | chr3:+6405972 | Cs3g05060 | intron | No | No |
| 6 | **T**T**A**TAT**G**TAGA**A**AAAGGAAA**AGG** | chr9:-15859965 | Cs9g16440 | intron | No | No |
| 7 | G**A**TTATATA**T**AGAA**GA**GAAA**TGG** | chr2:-30108859 |  | Intergenic | No | N.A. |
| 8 | GTTTATA**G**A**AGA**AAAGGAAA**AGG** | chr6:+18869249 | Cs6g18850 | intron | No | No |
| 9 | GTTT**G**TA**C**A**TG**GAAAGGAAA**AGG** | chr7:-7084189 |  | Intergenic | No | No |
| 10 | **C**TTTA**A**AT**TA**AGAAAGGAAA**AGG** | chr5:-32692617 | Cs5g30880 | intron | No | No |
| 11 | GT**A**TAT**GG**AGAGAAAG**A**AAA**AGG** | chr2:-4970243 | Cs2g08250 | utr | No | No |
| 12 | **AC**TTATAT**G**GAG**G**AAGGAAA**TGG** | chr4:-18596584 | Cs4g19110 | CDS | No | No |
|  | For CsALS: |  |  |  |  |  |
| 1 | **TT**GGT**G**CCTCGGAGG**G**TGAT**GGG** | chr9:-1639636 |  | Intergenic | No | No |

Note: N.A., not amplified
